# Surface Plasmon Resonance as a Characterization Tool for Lipid Nanoparticles Used in Drug Delivery

**DOI:** 10.3389/fchem.2020.605307

**Published:** 2021-01-07

**Authors:** Cecilia Yamil Chain, María Antonieta Daza Millone, José Sebastián Cisneros, Eduardo Alejandro Ramirez, María Elena Vela

**Affiliations:** Instituto de Investigaciones Fisicoquímicas Teóricas y Aplicadas (INIFTA- Universidad Nacional de La Plata (UNLP)- Consejo Nacional de Investigaciones Científicas y Técnicas (CONICET)), La Plata, Argentina

**Keywords:** lipid nanoparticles, drug carriers, Surface Plasmon Resonance, molecular target, protein corona

## Abstract

The development of drug carriers based in lipid nanoparticles (LNPs) aims toward the synthesis of non-toxic multifunctional nanovehicles that can bypass the immune system and allow specific site targeting, controlled release and complete degradation of the carrier components. Among label free techniques, Surface Plasmon Resonance (SPR) biosensing is a versatile tool to study LNPs in the field of nanotherapeutics research. SPR, widely used for the analysis of molecular interactions, is based on the immobilization of one of the interacting partners to the sensor surface, which can be easily achieved in the case of LNPs by hydrophobic attachment onto commercial lipid- capture sensor chips. In the last years SPR technology has emerged as an interesting strategy for studying molecular aspects of drug delivery that determines the efficacy of the nanotherapeutical such as LNPs' interactions with biological targets, with serum proteins and with tumor extracelullar matrix. Moreover, SPR has contributed to the obtention and characterization of LNPs, gathering information about the interplay between components of the formulations, their response to organic molecules and, more recently, the quantification and molecular characterization of exosomes. By the combination of available sensor platforms, assay quickness and straight forward platform adaptation for new carrier systems, SPR is becoming a high throughput technique for LNPs' characterization and analysis.

## 1. Introduction

Drug delivery has been improved over the years with continuous effort to develop new and more efficient carriers. Nowadays, drug carrier design points toward a non-toxic multifunctional nanoparticle (NP) that eludes the immune system and allows site-specific targeting, on-demand drug release and complete degradation of the carrier components (Choi and Han, 2018; Li et al., 2019; Yan et al., 2019; Zhao et al., 2019; Yeh et al., 2020).

Lipid-based nanoparticles (LNPs) bear the advantages for *in vivo* applications of being non- toxic and biodegradable (Puri et al., 2009) and they have probed their usefulness as vehicles for dermal, transdermal, mucosal, parenteral and ocular drug administration routes (Allen and Cullis, 2013; Desfrançois et al., 2018). Among them, phospholipid vesicles or “liposomes” were the first and so far most successful form of nanocarriers, with the larger number of approved formulations (Bozzuto and Molinari, 2015; Pattni et al., 2015; Bunker et al., 2016; Zylberberg and Matosevic, 2016; Li et al., 2019). Liposomes can be modified with a “stealth sheath,” e.g., with poly- ethylene glycol (PEG), to avoid the complement activation of the immune system. Another strategy to evade the immune system is to utilize natural vesicles such as exosomes (György et al., 2015; Ha et al., 2016; Wiklander et al., 2019). On the other hand, immunogenic properties can be exploited for targeting in virus-like particles (VLPs) that display effective cell entry properties due to their viral origin (Zdanowicz and Chroboczek, 2016; Rohovie et al., 2017). Nevertheless, all these vesicles lack of long-term storage stability and, if taken orally, they suffer a rapid degradation by stomach pH, bile salts or intestinal enzymes (Selvamuthukumar and Velmurugan, 2012). These difficulties and the need of compatible large-scale manufacturing lead to the development of solid lipid nanoparticles (SLNs) and, more recently, nanostructured lipid carriers (NLCs) (Naseri et al., 2015; Oner et al., 2020). NLCs incorporate small amounts of liquid lipids in the formulation diminishing matrix crystallization, increasing drug loading and preventing drug expulsion during storage and they have proved to be cytocompatible (Rodenak-Kladniew et al., 2019; Bueloni et al., 2020) and effective for different delivery routes.

**Figure 1:**
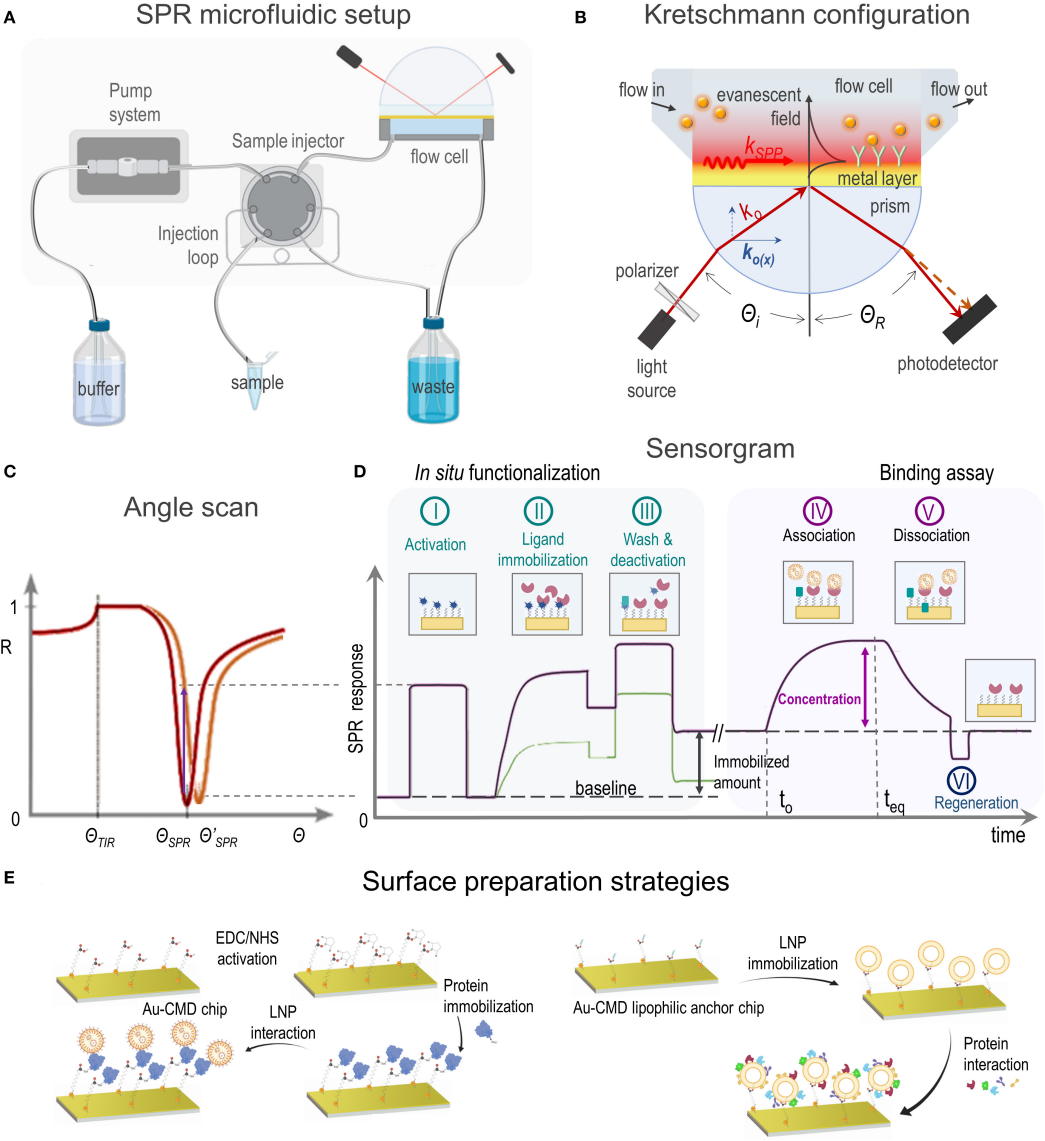
**(A)** SPR microfluidic setup, **(B)** SPR biosensor based on Kretschmann configuration showing incident light (k_o_) and SPP excitation principle, **(C)** SPR curve shift toward a higher angle (red to orange) due to a change in the refractive index, **(D)** sensorgrams showing SPR response vs. time during *in situ* ligand immobilization (steps I–III) and analyte binding assay (steps IV–VI) and **(E)** most common experimental strategies of SPR applications dealing with LNPs.

The adequate characterization of LNPs is crucial to obtain drug vehicles and to understand their behavior in biological systems. The characterization methods should focused on the LNP's parameters that determine their usefulness in nanotherapeutics: particle size and zeta potential, drug loading and drug release, stability and biomolecular interactions, among others (Mehnert and Mäder, 2012). Most of the conventional methods to study biomolecular interactions require labeling, such as ELISA, fluorescence techniques or MicroScale Thermophoresis (MST) (Jerabek-Willemsen et al., 2014). Among label-free techniques, binding affinity can be assessed by Isothermal Titration Calorimetry (ITC) (Duff et al., 2011) or Biolayer Interferometry (BLI) (Weeramange et al., 2020) but, as they lack from dynamic flow conditions, kinetic parameters cannot be determined. Surface Plasmon Resonance (SPR) spectroscopy is a label free optical technique capable of real-time measuring through changes in the refractive index (RI) in the vicinity of a metal surface. To do so, one binding partner (ligand) is immobilized on a sensor chip while the free counterpart (analyte) from a sample solution is injected through a microfluidic setup (Figure 1A). Although similar information is provided by the quartz crystal microbalance (QCM), in this case the obtained data depends both on the analyte binding and on the water displacements that can occur due to the interaction, affecting the obtained results (Tonda-Turo et al., 2018).

The aim of this review is to outline the state of the art in SPR sensing of LNPs, describing its usefulness and challenges in the framework of drug delivery research.

## 2. Surface Plasmon Resonance

### 2.1. Principle

In order to achieve the SPR phenomenon, a material that exhibits a free electron behavior is required, as it occurs with the conduction band electrons in metals (Maier, 2007). Plasmons are quantized waves of the collective movement of electrons resulting from the interaction with photons from a *p*-polarized light source. The propagation of plasmons, now surface plasmon-polaritons (SPPs), produces alternating positive and negative regions that in contact with a lower RI medium result in a confined evanescent field, that decays exponentially in the perpendicular direction at both sides of the interface (Figure 1B). Small changes in RI within the evanescent field will greatly alter the propagation properties of SPPs, reason why this phenomenon drew attention as an analytical technique.

The resonant condition, where the largest number of photons can excite SPPs, will be reached when the light wavevector in the propagation direction [k_o(x)_] matches the wavevector of the SPPs (k_SPP_). There are different strategies to achieve this matching, the most widely employed method in SPR instruments is the Kretschmann configuration (Figure 1B) where SPP excitation is attained through total internal reflection (TIR) (Schasfoort, 2017). Depending on the incident angle (Θ_i_), the k_o(x)_ light component is absorbed by SPPs, resulting in a drop of reflectivity (R) (Figure 1C). At a given RI in the medium, Θ_*SPR*_ allows the maximum light absorption by SPPs and the minimum reflected light. As RI changes, i.e., by molecules in a solution, k_SPP_ is modified and a different Θ_*SPR*_ is required to fulfill the new resonance condition. SPR measurements are usually carried out at a fixed Θ_i_ and changes expressed as SPR response (either ΔR or ΔΘ) are registered as a function of time (Figure 1D), yielding curves known as sensorgrams.

### 2.2. Measurement Data and Analysis

SPR sensors chips are usually gold coated glass plates. The optimal gold layer thickness of ~ 50 nm allows to achieve high sensitivity (Fontana, 2006) and the glass side is coupled to a prism through a RI matching fluid or polymer (Figure 1B). A flow cell is placed upon the gold surface enclosing one or more flow channels. The microfluidic SPR setup allows to infuse a running buffer and a sequential injection of small volume (50–1,000 μL) sample solutions that will interact with the sensor platform (Figure 1A). The adequate choice of the flow rate is critical to avoid mass transport limitations or shear stress effects.

Platform design should be optimized to prevent steric hindrance that can affect binding events. Depending on the application, the gold layer needs to be modified (section 3) and a biocompatible organic layer (thiol self-assembled monolayer—SAM- or polymer), eventually exhibiting anchor points for immobilization, is placed onto the surface (Gedig, 2017). Ligand immobilization can take place either *ex situ* or *in situ*, in the latter case the immobilized amount can be quantified (Figure 1D; Albers and Vikholm-Lundin, 2011). For immobilization by covalent attachment, crosslinker reagents are injected in the first place to activate the surface (Figure 1D, step I) and the ligand solution is subsequently passed over for a time period that allows conjugation (Figure 1D, step II). On the other side, a ligand solution can be directly injected without previous activation (Figure 1D, step II) if immobilization is based on physical attachment, i.e., by hydrogen bonding or hydrophobic forces. Finally, a washing procedure (Figure 1D, step III) is employed to either remove weakly bound material and/or deactivate the reactive functional groups generated in step I.

Once the surface is ready, a solution containing the analyte is injected allowing association (Figure 1D, step IV) and registering afterwards the dissociation (Figure 1D, step V). Before the next analyte assay the surface must be regenerated (Figure 1D, step VI) with the mildest solution that releases the analyte without damaging the immobilized ligand, i.e., diluted acids or bases, detergents, etc. As the amount of the SPR response at the steady state (t_eq_) is related to the analyte concentration and possible matrix interferents, control experiments must be carefully designed in order to avoid data misinterpretation. Moreover, to minimize unspecific adsorption, a blocking step to reduce non-specific binding can also be included before the assay, e.g., by using a well-known protein solution.

## 3. Surface Preparation Strategies

Since SPR studies of LNPs cover a great variety of applications, several immobilization methods have been utilized, mainly based on covalent immobilization or hydrophobic attachment of one of the interacting entities onto organic layer covered- gold sensor surfaces (Figure 1E). High affinity capture of ligands by a specific binding molecule and adsorption onto bare gold sensor chips have also been reported. In this section, a brief description of each immobilization method utilized in SPR studies of LNPs is presented. Reference papers corresponding to each subsection are listed in Table 1.

**Table 1:** Summary of SPR studies on LNPs: research area, subject of study, immobilized ligand and analyte in solution, surface preparation strategy, and reference papers.

**Lipid nanoparticle**	**Research area**	**Subject of study**	**Immobilized ligand/analyte in solution**	**Sensor chip and surface immobilization chemistry**	**References**
Liposomes	Molecular interactions involved in drug delivery	Interaction with biological targets (4.1.1)	Target protein/LNP	Au-CMD chip, amide coupling (3.1)	Laukkanen et al., 1994; Nielsen et al., 2002; Terada et al., 2007; Mizrahy et al., 2011; Etzerodt et al., 2012; Ding et al., 2015; Shi et al., 2015a,b; Xiang et al., 2015; Gregori et al., 2017; Huang et al., 2019
				Au-alginate chip, amide coupling (3.1)	Gobbi et al., 2010; Mourtas et al., 2011
				Au chip, biotinylated thiol layer (3.2)	Viitala et al., 2012
				Au-CMD chip, amide coupling, streptavidin (3.2)	Al-Ahmady et al., 2014
			LNP/target protein	Au 1-octadecanethiol SAM chip (3.3)	Tamiaki et al., 2006
				Au chip, 11-mercapto 1- undecanol SAM (3.3)	Sandoval-Altamirano et al., 2017
			Cell membrane model/LNP	Au lipid- capture chip (3.3)	Cai et al., 2014; Wang et al., 2014
			Cell/LNP	Au-CMD chip, amide coupling (3.1)	Guo et al., 2014
			Bacterial biofilm/LNP	Au chip, incubation with diluted bacterial culture suspensions	Sugano et al., 2016
		Interaction with serum proteins (4.1.2)	LNP/protein	Au lipid- capture chip (3.3)	Akita et al., 2015; Shibata et al., 2015; Kari et al., 2017
				Au chip, 1-octadecanethiol SAM (3.3)	Malmsten, 1999; Efremova et al., 2000
				Au chip, biotinylated thiol layer, streptavidin (3.2)	Meierhofer et al., 2010
			Protein/LNP	Au-CMD chip, amide coupling (3.1)	Crielaard et al., 2011
				Au-alginate chip, amide coupling (3.1)	Canovi et al., 2012
		Interaction with ECM matrix (4.1.3)	Protein/ LNP	Au-CMD chip, amide coupling (3.1)	Wadajkar et al., 2019
	Obtention and characterization of LNP's formulations	Interaction between components of the formulations (4.2.1)	LNP/protein	Au lipid- capture chip (3.3)	Rauscher et al., 2014; Skyttner et al., 2019
			LNP/PEG	Au chip (3.4)	Zhao et al., 2010
			Protein/LNP	Au-CMD chip, amide coupling (3.1)	Yatuv et al., 2009
		Response to organic molecules (4.2.2)	LNP/detergent	Au-lipid capture chip (3.3)	Shibata et al., 2012
			LNP/ glucose	Au chip (3.4)	Seong et al., 2003
Hybrid NPs	Obtention and characterization of LNP's formulations	Interaction between components of the formulations (4.2.1)	Hybrid NP/targeting peptide	Au lipid- capture chip (3.3)	Soman et al., 2008; Pan et al., 2011
			Liposome/polymeric NP	Au lipid- capture chip (3.3)	Gao et al., 2014
	Molecular interactions involved in drug delivery	Interaction with biological targets (4.1.1)	Hybrid NP/cell	Au lipid. capture chip (3.3)	Soman et al., 2009
Exosomes	Quantification and molecular characterization of exosomes	Quantification of exosomes (4.3)	Specific biotinylated antibody/exosome	Au chip, biotinylated thiol layer, streptavidin (3.2)	Rupert et al., 2016
			Streptavidin/biotinylated exosome	Au chip, biotinylated thiol layer (3.2)	Rupert et al., 2014
		Identification of exosomal proteins (4.3)	Specific biotinylated antibody/exosome	Au chip, thiolated PEG /streptavidin (3.2)	Im et al., 2017
SLN	Molecular interactions involved in drug delivery	Interaction with biological targets (4.1.1)	Protein/LNP	Au-alginate chip, amide coupling (3.1)	Gobbi et al., 2010
		Interaction with serum proteins (4.1.2)	Protein/LNP	Au chip, 11-mercaptoundecanoic acid SAM, amide coupling (3.1)	Di Ianni et al., 2017
NLC	Molecular interactions involved in drug delivery	Interaction with biological targets (4.1.1)	Protein/LNP	Au-CMD chip, amide coupling (3.1)	Rafique et al., 2019
VLP	Molecular interactions involved in drug delivery	Interaction with biological targets (4.1.1)	Cell membrane model/LNP	Au lipid- capture chip (3.3)	Jedynak et al., 2018

*Parentheses indicate subsections where subjects of study and surface immobilization chemistries are described*.

### 3.1. Covalent Immobilization

Among conjugation techniques to immobilize ligands to SPR surfaces, amide linkage (Hermanson, 2013) is the most employed strategy. Functional carboxylic groups are included in carboxymethyl dextran (CMD) or alginate coated commercial gold sensor chips or they can be obtained from bare sensor surfaces adequately covered with thiol SAMs (Table 1) based on the robust covalent bond that is established between S and Au (Vericat et al., 2010).

Although covalent coupling easily provides stable ligand immobilization to the sensor surface, it may modify active sites of proteins potentially affecting the analyte binding activity. In cases where the covalent immobilization of the ligand is unsuitable, capturing methods provide an alternative approach.

### 3.2. Capturing Approaches

Ligand immobilization based on high affinity streptavidin- biotin capture has also been reported in SPR studies dealing with LNPs. The surface preparation relies on the adequate attachment of streptavidin onto the sensor surface either through reaction with biotinylated alkanethiols (Meierhofer et al., 2010; Rupert et al., 2016), thiolated PEG (Im et al., 2017) or amide coupling (Al-Ahmady et al., 2014) and in the previous ligand's conjugation to biotin (Hermanson, 2013).

### 3.3. Hydrophobic Attachment

Immobilization of LNPs to SPR chips through hydrophobic interactions can be achieved by alkane chains incorporated in a polymeric coating, commercially referred to as “Au- lipid capture chips” or prepared by covering bare sensor chips with alkanethiol- SAMs (Table 1).

Most of SPR applications focused on surface attached LNPs are based on Au- lipid capture chips coated with CMD and functionalized with lipophilic substituents (Table 1), as these platforms yield the immobilization of “intact” LNPs (Figure 1E, right; Hodnik and Anderluh, 2013). On the other side sensor surfaces coated with alkanethiol groups, either obtained commercially (Tamiaki et al., 2006) or prepared by chemical modification of bare gold sensor chips (Malmsten, 1999; Efremova et al., 2000), have also been used to immobilize LNPs, with the limitation that NPs fuse to the surface generating a lipid monolayer onto the alkanethiol (Hodnik and Anderluh, 2013).

### 3.4. Physical Adsorption

Some SPR applications dealing with immobilized LNPs are based on simple physical attachment to SPR bare gold chips (Table 1) as some biomolecules show a strong spontaneous adsorption on gold surfaces (Hodnik and Anderluh, 2013). Nevertheless, the reorganization or uncontrolled exchange of the adsorbed entities to attain the most favorable thermodynamic state have been reported (Hodnik and Anderluh, 2013) which can result in unreliable assays.

## 4. Applications of SPR-Based Biosensors in the Study of LNPs

SPR-based sensors are increasingly used to study a variety of LNPs such as liposomes, SLNs, NLCs, VLPs, exosomes and hybrid systems. In this section, examples of applications of SPR biosensing on drug LNPs' carriers in different areas of nanotherapeutics research are presented.

### 4.1. LNPs' Interactions Involved in Drug Delivery

#### 4.1.1. Interaction With Biological Targets

One of the major challenges of nanotherapeutics is to selectively deliver NPs to the desired biological target. With this aim, NPs are functionalized with adequate targeting ligands resulting in decorated nanovehicles with enhanced capacity to direct selective binding. Analyses of interactions between LNPs and their biological molecular targets is a well-established research area of SPR, either by using isolated proteins, cell membranes or entire cells as target models (Table 1).

SPR experiments have been defining in the obtention of decorated liposomes with high affinity to amyloid-β peptide (Gobbi et al., 2010; Mourtas et al., 2011; Gregori et al., 2017), contributing to the development of very promising vectors for the targeted delivery of potential new diagnostic and therapeutic molecules for Alzheimer's disease. SPR sensing based on surface immobilization of the target protein onto Au- CMD chips (Figure 1E, left) has also contributed to the elucidation of the interplay of decorated liposomes or SLNs and membrane proteins that are overexpressed in malignant tissues (Nielsen et al., 2002; Terada et al., 2007; Mizrahy et al., 2011; Shi et al., 2015a; Huang et al., 2019), in disease-supporting macrophages (Etzerodt et al., 2012; Rafique et al., 2019) or in T-cells involved in autoimmune diseases (Ding et al., 2015). Moreover, SPR studies based on immobilized liposomes (Figure 1E, right) have shed light on the complex mechanism for the interaction of lectins with glycoliposomes specially designed to target sugar-binding proteins (Tamiaki et al., 2006; Sandoval-Altamirano et al., 2017).

The interplay between LNPs and cell membranes or entire cells as biological targets have also been investigated by SPR (Table 1). In this regard, SPR has been applied to study the interaction of liposomes in solution and immobilized bacterial biofilms (Sugano et al., 2016) or tumoral cell lines (Guo et al., 2014), and a work describing the application of SPR to assess the interaction of immobilized hybrid NPs and eukaryotic cells in solution has been reported (Soman et al., 2009).

#### 4.1.2. Interaction With Serum Proteins

It is well-known that the interaction of drug nanocarriers with serum proteins can alter the pharmacokinetics of the nanovehicles either affecting the cellular uptake or the clearance of the particles by the immune system (Pearson et al., 2014). SPR has been applied to study the interplay between NPs and isolated serum proteins in order to optimize LNP's formulation design (Table 1). SPR has contributed to study fibrinogen, human serum albumin and bovine pancreatic trypsin inhibitor adsorption onto both neutral and negatively charged PEG-decorated liposomes (Efremova et al., 2000). Interestingly, the observed reduction in protein adsorption as PEG densities in the nanovehicles increases agreed with theoretical predictions. These results suggest that SPR studies could contribute to establish the physical basis of the different interactions of LNPs with proteins and cells. Moreover, particular proteins of the NPs' corona were identified by means of SPR experiments on LNPs preincubated with serum (Canovi et al., 2012) and the real-time protein corona formation was followed on surface-immobilized NPs (Kari et al., 2017).

#### 4.1.3. Interaction With Tumor ECM

Therapeutic efficacy of drug nanovehicles for cancer applications is significantly impaired by limited tumor tissue penetration due to a physical barrier formed by extracellular matrix (ECM) proteins. In this regard, SPR has been recently expanded as a method to examine the interfacial properties of liposomes, by analyzing their binding properties toward surface immobilized tumor ECM proteins as a surrogate for their ability to penetrate solid tumors (Wadajkar et al., 2019).

### 4.2. Obtention and Characterization of LNPs' Formulations

#### 4.2.1. Interaction Between Components of the LNPs' Formulation

Stability, targeting specificity and drug release efficiency of nanosized drug carriers can be improved by nanovehicle's surface functionalization. In this sense, SPR has contributed to the optimization of LNP- based drug delivery systems by providing a rapid screening method to assess the interaction between LNP and potential binding molecules to be included in the final NP formulation (Table 1). Soman et al., by way of illustration, incorporated the peptide melittin in the outer lipid monolayer of perfluorocarbon (PFC) NPs and demonstrated the tight binding of this potential cancer chemotherapeutic with the nanocarriers from the SPR data (Soman et al., 2008).

#### 4.2.2. LNPs' Response to Organic Molecules

LNPs can be easily captured on SPR chips by means of lipophilic anchors (Del Vecchio and Stahelin, 2016) as described in section 3.3 and the obtained sensor surfaces can be used to investigate the interplay of the immobilized nanovehicles and organic molecules in aqueous solutions (Seong et al., 2003; Shibata et al., 2015). For instance, Shibata et al. utilized SPR to study the interaction of different detergents (two bile salts and Triton X-100) and PEGylated liposomes that were immobilized to the surface of a lipid- capture chip (Shibata et al., 2012). The authors observed that the detergents were either bound to or partitioned into lipid bilayers and they subsequently solubilized and dissociated from the chip. These results suggest that SPR can provide an automatized method to simply address the solubilization and interaction of detergents with LNPs.

### 4.3. Quantification and Molecular Characterization of Exosomes

The increasing interest of the scientific community in using exosomes as drug nanovehicles has generated a growing need for sensitive methods capable of quantifying and characterizing these nanosized cell- secreted vesicles. Recent SPR reports in the field of exosome's investigation are based on the immobilization of specific antibodies against exosomal proteins onto the sensor surface and the measuring of SPR response as exosomes in solution are injected into the setup (Table 1). A thorough review of the use of SPR as a method for sensitive detection and molecular characterization of exosomes can be found in the literature (Rojalin et al., 2019).

## 5. Conclusions

SPR technology has emerged as an interesting strategy for studying different aspects of LNPs intended to deliver bioactive molecules, from the physicochemical characterization and quantification of lipid- based drug carriers to the study of the interaction of nanovehicles with the biological entities that they will encounter in therapeutic applications.

Although SPR technique is a well-established tool for studying molecular interactions, the experiment's reliability is based on the adequate immobilization of one of the interacting partners on the sensor surface and in the absence of interferences that could affect the resulting signals. Concerning LNPs, chemical constraints that may appear in the sensor surface preparation can be easily overcome as lipid based nanovehicles can be directly immobilized onto commercial lipid- capture chips by hydrophobic attachment.

Regardless of the numerous reports presented in this review, SPR still remains as an underexploited technique in the field of LNP development and analysis. Measurement's simplicity and quickness should position SPR as a high-throughput technique for LNPs' characterization, providing preliminary information about biomolecular interactions that LNPs experiment in therapeutic applications and allowing to tune formulations before *in vivo* experiments. Finally, given the universal SPR detection principle, assays can be straight forward adapted to new drug carriers, a trend that with the incorporation of VLPs, exosomes and hybrid systems is in continuous growth.

## Author Contributions

CYC, MADM, JSC, EAR, and MEV wrote and revised the manuscript and approved it for publication. All authors contributed to the article and approved the submitted version.

## Conflict of Interest

The authors declare that the research was conducted in the absence of any commercial or financial relationships that could be construed as a potential conflict of interest.
